# Prognostic significance of C-reactive protein in patients with cervical cancer: a meta-analysis

**DOI:** 10.3389/fonc.2023.1232409

**Published:** 2023-09-01

**Authors:** Sheng Yang, Zongxin Zhang, Linglong Shen

**Affiliations:** ^1^ Clinical Laboratory, Huzhou Maternity and Child Health Care Hospital, Huzhou, Zhejiang, China; ^2^ Clinical Laboratory, Huzhou Central Hospital, Affiliated Central Hospital of Huzhou University, Huzhou, Zhejiang, China

**Keywords:** C-reactive protein, cervical cancer, meta-analysis, prognosis, evidence-based medicine

## Abstract

**Background:**

Numerous studies have investigated the significance of pretreatment C-reactive protein (CRP) levels for determining the prognosis of cervical cancer (CC). The results of these studies, however, have been inconsistent. The present meta-analysis, therefore, focused on identifying the exact relationship of CRP levels with CC prognoses.

**Methods:**

We searched the following databases from their inception until April 18, 2023: PubMed; Web of Science; Embase; and Cochrane Library. From the search results, we estimated the significance of CRP levels in determining the prognosis of CC, based on combined hazard ratios (HRs) and relevant 95% confidence intervals (CIs).

**Results:**

The present meta-analysis included 12 studies, encompassing 2,204 patients. Based on combined data, an increased CRP level was significantly related to an unfavorable overall survival (OS) of patients with CC (HR = 1.63; 95% CI = 1.36–1.95; *P* < 0.001). Moreover, an increased CRP level was significantly associated with shortened progression-free survival (PFS) in patients with CC (HR = 1.68; 95% CI = 1.39–2.03; *P* < 0.001). According to the subgroup and sensitivity analyses, CRP level was a reliable factor in determining CC prognoses.

**Conclusion:**

Based on the results of our present analyses, increased CRP levels were significant predictors of poor OS and PFS in patients with CC. CRP level, therefore, could be an independent and inexpensive factor for determining the prognosis of patients with CC in clinical settings.

**Systematic review registration:**

INPLASY, identifier INPLASY202360074.

## Introduction

Cervical cancer (CC) ranks the fourth most common cancer in women; besides, it is also the fourth frequent cause of cancer death among women globally (1). There were 604,127 new cases of CC and 6 341,831 deaths due to CC in 2020 worldwide (1, 2). The primary etiological factor for CC is chronic human papillomavirus (HPV) infection (3, 4), and surgical resection is the preferred treatment for patients diagnosed with International Federation of Gynecology and Obstetrics (FIGO) stages ≤ IIA, while chemoradiation is recommended for patients diagnosed with higher stages (3, 5). It is estimated, however, that 20–25% of patients with CC will experience a recurrence after completing their primary treatment, and that locally advanced CC has a poor 5-year survival rate (6). To some extent, the lack of efficient prognostic markers may have a correlation to poor CC survival. Therefore, establishing reliable and easily available biomarkers for determining the prognosis of patients with CC would aid the development of treatment strategies.

A growing body of evidence suggests that inflammation is involved in the pathogenesis and development of solid tumors (7, 8). When infection, tissue injury, trauma, neoplastic growth, or surgery interferes with the homeostasis of an organism, an acute-phase response (APR) is immediately triggered (9). C-reactive protein (CRP), a sensitive and well-recognized systemic inflammatory marker produced by the liver in response to factors such as interleukin (IL)-6, IL-1, along with tumor necrosis factor-α (TNF-α) (10). CRP is an acute phase reactant that is widely considered to be a marker of both acute and chronic inflammation (11). CRP has several well-defined functions, including acting as a pattern recognition receptor with calcium-sensitive binding pockets for ligands expressing phosphocholine (PC) moieties (12). CRP can also bind to activated cell membranes in which the PC groups on phospholipids become accessible when diacylphospholipids are hydrolyzed into monoacylphospholipids (12). In addition to being found in serum, CRP has recently been found to exist in an array of cyclic pentameric discs. The effects of CRP on the behavior of cells and the microenvironment are, therefore, dependent on its structural configuration. Pentameric CRP (pCRP) binds to PC on the surface of the cell, causing it to dissociate into its distinct monomeric isoform (mCRP), which subsequently reduces its aqueous solubility (13). It has been suggested that CRP levels are an effective factor in determining the prognosis of various cancers, including esophageal cancer (14), renal cell carcinoma (RCC) (15), prostate cancer (16), and gastric cancer (17). The relationship between CRP levels and survival outcomes in patients with CC has been extensively investigated; however, no consistent results have been reported thus far (18–29). Elevated CRP levels have been reported to be a significant prognostic factor for CC in some studies (18, 20, 22), while others have demonstrated a non-significant association between CRP levels and CC prognosis (19, 24). Given these inconsistent results, we aimed to comprehensively search relevant articles in the present meta-analysis to quantitatively identify the true relationship of CRP levels and CC prognoses.

## Materials and methods

### Study guidelines

The present meta-analysis was conducted according to the Preferred Reporting Items for Systematic Reviews and Meta-Analyses (PRISMA) (30), and was registered with the International Platform of Registered Systematic Review and Meta-analysis Protocols (INPLASY) platform. The registration number of this meta-analysis on INPLASY is INPLASY202360074.

### Ethnics statement

The present meta-analysis did not require the involvement of an institutional review board or ethics committee, as all of the data acquired were derived from previously published studies.

### Literature search

In the present study, we thoroughly searched the PubMed, Web of Science, Embase, and Cochrane Library databases from their inception until April 18, 2023, for the following keywords/search terms: (C-reactive protein OR CRP OR c-reactive protein) AND (cervical cancer OR cervical carcinoma OR uterine cervix cancer OR cervical neoplasm OR cervix cancer). Only English studies were considered. After the initial search, the references from each of the included articles were manually searched to identify additional relevant articles.

### Eligibility criteria

The inclusion criteria were as follows: (i) CC diagnosis confirmed based on pathology; (ii) studies explored the between serum CRP levels and any survival outcomes of patients with CC; (iii) available hazard ratios (HRs) and associated 95% confidence intervals (CIs) for prognoses from studies or calculable data based on the information provided in the articles; (iv) a cut-off value was defined with which to determine low/high CRP levels; and (v) studies were published in English. The exclusion criteria were as follows: (i) articles formatted as reviews, meeting abstracts, case reports, comments, or letters; (ii) patients with new infections, or chronic infection diseases, autoimmune diseases, organ dysfunctions, hematologic diseases, and patients with another type of tumor; (iii) articles involved animal studies; and (iv) duplicate articles.

### Extraction of data and evaluation of study quality

Two researchers (SY and ZZ) independently screened all eligible studies and extracted the information required from each, which included the following: first author; publication year; country; sample size; study duration; age; FIGO stage; treatment; CRP level cut-off; cut-off determination method; survival endpoints; follow-up; survival analysis type; and HRs with 95% CIs. All disagreements were resolved by reaching a consensus after discussion with a third reviewer (LS). The overall survival (OS) was selected as the primary outcome, and progression-free survival (PFS) as the secondary outcome. Additionally, we utilized the Newcastle-Ottawa Scale (NOS) score to evaluate the quality of the included articles (31). The NOS evaluates studies based on 3 factors cohort selection; comparability; and results. A NOS score ≥ 6, based on a 0–9 point scale, suggests high-quality research.

### Statistical analysis

Pooled HRs and 95% CIs were calculated to estimate the significance of CRP levels in determining the prognosis for patients with CC. In general, a combined HR > 1 with a 95% CI not overlapping 1 indicated a significant association with poor prognosis, while a combined HR < 1 with a 95% CI not overlapping 1 indicated a better prognosis. Inter-study heterogeneity was evaluated using Cochran’s Q-test (32) and I^2^ statistics (33). I^2^ was used to quantify the degree of heterogeneity among the studies, as follows: I^2^ < 25%, low degree; 25–75%, moderate degree; and > 75%, high degree of heterogeneity (33, 34). To analyze the pooled data, we used two different computational models, based on the traits of the included studies, and the cut-off for significant heterogeneity was set at I^2^ > 50% (35–38). When high heterogeneity was determined based on I^2^ > 50% and Q-test P < 0.10, and a random-effects model (REM; DerSimonian-Laird method) was used (39); otherwise, a fixed-effects model (FEM; Mantel-Haenszel method) was used (40). Subgroup analyses of OS and PFS were conducted to identify possible sources of heterogeneity, and we conducted a sensitivity analysis by removing one article at a time, in order, to evaluate the robustness of the combined results. Funnel plots and Begg’s test (41) were utilized to evaluate publication bias. All statistical analyses were conducted using Stata software version 12.0 (Stata Corporation, College Station, TX, USA), with P < 0.05 indicating a significant difference.

## Results

### Included literature

**Figure 1:**
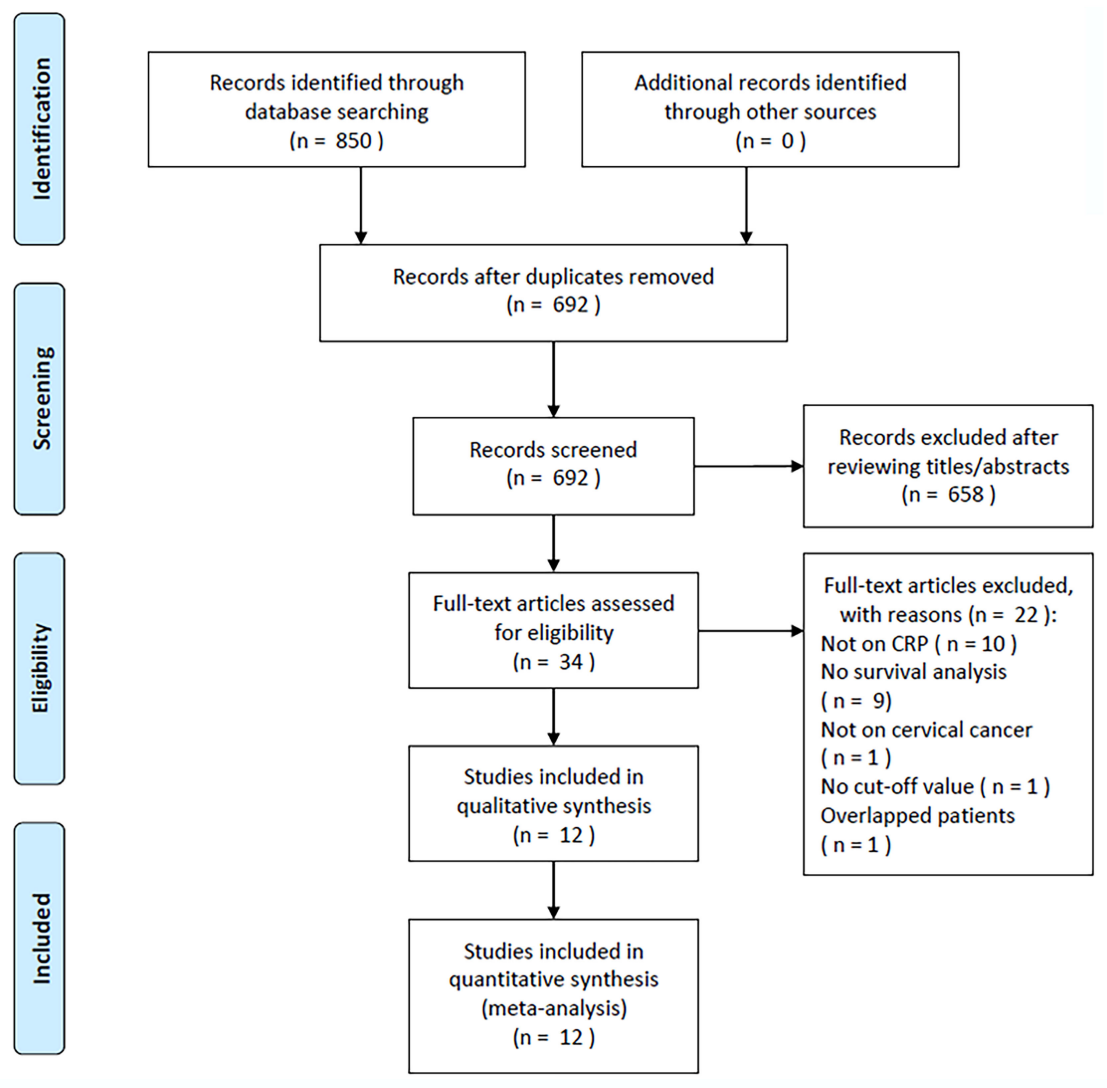
PRISMA flowchart of the established screening strategy.

During the initial literature search, 850 relevant articles were identified, and after the removal of duplicate studies, 692 studies remained (**Figure 1**). After title and abstract screening, 658 articles were excluded because they were irrelevant studies or animal studies, the full texts of the remaining 34 articles were read, and 22 articles were further eliminated, for the following reasons: irrelevance to CRP (n = 10); non-available survival analysis data (n = 9); irrelevance to CC (n = 1); no identified cut-off value (n = 1); or overlapping cases (n = 1). In total, we included 12 articles in the present study, encompassing 2,204 cases (**Figure 1**, **Table 1**) (18–29).

**Table 1:** Basic characteristics of included studies in this meta-analysis.

Author	Year	Country	Sample size	Study period	Age (year) Median(range)	FIGO stage	Treatment	CRP cut-off value (mg/L)	Cut-off determination	Survival outcome	Follow-up (month) Median(range)	Survival analysis	NOS score
Polterauer, S.	2007	Austria	214	1995-2006	50	I-IV	Mixed	5	Median value	OS, PFS	29.4	Multivariate	7
Nakamura, K.	2015	Japan	32	2005-2014	52.6 (25–78)	Recurrent	CCRT	7	ROC curve	OS	6.6(1.4-34.1)	Univariate	7
Xiao, Y.	2015	China	238	2004-2011	54(34-70)	I-IV	CCRT	10	ROC curve	OS, PFS	42(15-91)	Univariate	8
Bodner-Adler, B.	2016	Austria	46	2005-2015	51(24-60)	I-IV	Mixed	5	ROC curve	OS	1-167	Multivariate	7
He, X.	2018	China	229	2007-2009	44(28-79)	I-IV	Mixed	10	ROC curve	OS	1-80	Univariate	8
Wang, W. J.	2019	China	110	2012-2014	51.5(25-70)	I-II	Mixed	2.62	Median value	OS, PFS	1-36	Multivariate	8
An, Q.	2020	China	278	2010-2017	45.5	I-II	Mixed	6.1	ROC curve	OS, PFS	1-60	Univariate	7
Wang, H.	2020	China	150	2013-2015	59(24-75)	I-III	CCRT	5	Median value	OS	39(8-77)	Univariate	8
Bakir, M. S.	2021	Turkey	243	2002-2020	48(28-84)	I-IV	Mixed	9.59	ROC curve	OS	70.2(0.5-211.2)	Univariate	8
Taguchi, A.	2021	Japan	89	2004-2015	67	Recurrent	Radiotherapy	7.35	Median value	OS	16.4	Univariate	7
Li, Y.	2022	China	460	2011-2019	49.8	I-III	CCRT	2.6	ROC curve	OS, PFS	1-90	Univariate	8
Zheng, X.	2023	China	115	2020-2022	54(32-70)	Recurrent/ metastatic	Anti-PD-1	3.08	ROC curve	PFS	11.3(2.2-28.7)	Multivariate	9

CRP, C-reactive protein; CCRT, concurrent chemoradiotherapy; ROC, receiver operating characteristic; OS, overall survival; PFS, progression-free survival; FIGO, International Federation of Gynecology and Obstetrics; NOS, Newcastle-Ottawa Scale; PD-1, programmed death-1.

### Study features

**Table 1** summarizes the basic characteristics of the present study. All of the eligible studies were published between 2007 and 2023, with 7 published in China (20, 22–25, 28, 29), 2 in Austria (18, 21), 2 in Japan (19, 27), and 1 in Turkey (26). Sample sizes ranged from 32 to 460 (median, 182) participants. Of the 12 included studies, 5 recruited patients diagnosed with FIGO stages I–IV (18, 20–22, 26), 2 with stages I–II (23, 24), and 2 with stages I–III (25, 28), while 3 included recurrent/metastatic tumor cases (19, 27, 29). The threshold CRP value was 2.6–10 (median, 5.55) mg/L, and 8 articles determined the cut-off value using receiver operating characteristic (ROC) analysis (19–22, 24, 26, 28, 29), while 4 studies utilized the median value of that individual study (18, 23, 25, 27). Of the 12 articles, 11 mentioned the significance of CRP in predicting OS (18–28), and 6 investigated the association between CRP and PFS in patients with CC (18, 20, 23, 24, 28, 29). HRs, together with the relevant 95% CIs, were collected through univariate regression in 9 articles (19, 20, 22, 24–28) and multivariate analysis in 4 (18, 21, 23, 29). The NOS scores all ranged from 7–9 (median, 8), indicating high quality articles (**Table 1**).

### Significance of CRP in predicting OS

**Figure 2:**
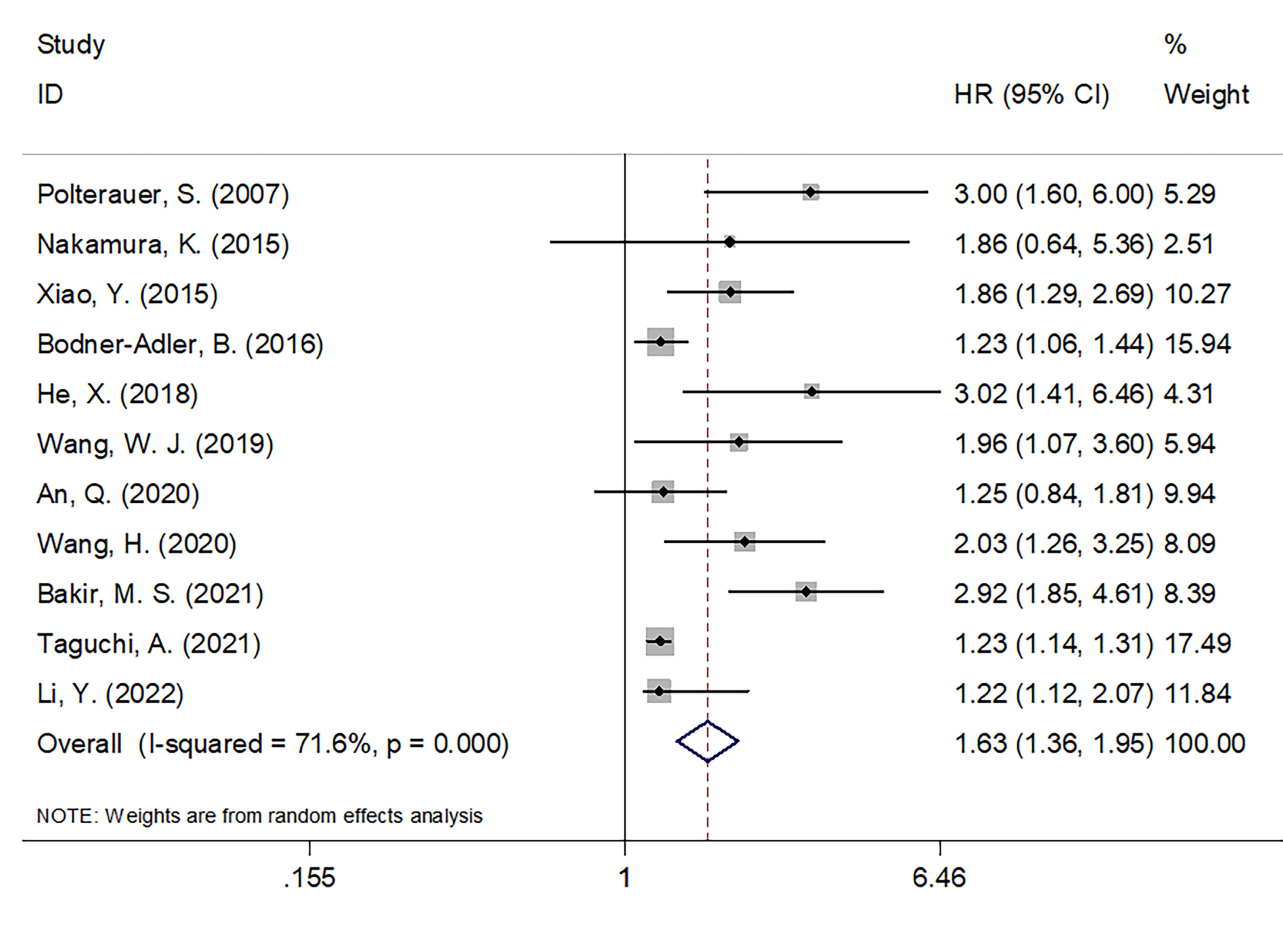
Forest plots of the prognostic role of CRP for OS in patients with CC.

A total of 11 articles, encompassing 2,089 patients (18–28), presented data on the relationship between the CRP value and OS in patients with CC. REM was utilized in these articles because significant heterogeneity was detected (I^2 = ^71.6%; P < 0.001). In **Table 2** and **Figure 2**, as seen based on the pooled data, increased CRP expression was significantly tied to extremely poor OS in patients with CC (HR = 1.63; 95% CI = 1.36–1.95; *P* < 0.001). Subgroup analyses were conducted based on various factors. As seen in **Table 2**, subgroup analysis results indicated that elevated levels of serum CRP were still a significant marker for predicting OS in patients with CC regardless of sample size, country, FIGO stage, treatment, CRP cut-off, cut-off determination, or survival analysis type (all, *P* < 0.05).

**Table 2:** Subgroup analysis of prognostic role of CRP for OS in patients with CC.

Subgroups	No. of studies	No. of patients	Effects model	HR (95%CI)	p	Heterogeneity	
						I^2^(%)	Ph
Total	11	2,089	REM	1.63(1.36-1.95)	<0.001	71.6	<0.001
Country							
China	6	1,465	FEM	1.57(1.32-1.86)	<0.001	45.9	0.100
Non-China	5	624	REM	1.61(1.24-2.10)	<0.001	80.7	<0.001
Sample size							
<200	5	427	FEM	1.25(1.17-1.33)	<0.001	42.1	0.141
≥200	6	1,662	REM	1.92(1.36-2.71)	<0.001	71.6	0.003
FIGO stage							
I-IV	5	970	REM	2.14(1.38-3.31)	0.001	82.8	<0.001
I-II/I-III	4	998	FEM	1.42(1.16-1.74)	0.001	35.2	0.201
Recurrent/ metastatic	2	121	FEM	1.23(1.15-1.32)	<0.001	0	0.446
Treatment							
CCRT	4	880	FEM	1.56(1.27-1.92)	<0.001	35.2	0.201
Mixed	6	1,120	REM	1.94(1.33-2.83)	0.001	78.3	<0.001
Radiotherapy	1	89	–	1.23(1.15-1.32)	<0.001	–	–
Cut-off value							
≤5 mg/L	5	980	REM	1.60(1.20-2.13)	0.001	65.7	0.020
>5 mg/L	6	1,109	REM	1.77(1.26-2.49)	0.001	78.4	<0.001
Cut-off determination							
ROC curve	7	1,526	REM	1.64(1.25-2.14)	<0.001	70.3	0.003
Median value	4	563	REM	1.83(1.18-2.83)	0.007	76.9	0.005
Survival analysis							
Multivariate	3	370	REM	1.80(1.03-3.14)	0.037	75.9	0.016
Univariate	8	1,719	REM	1.67(1.30-2.14)	<0.001	73.9	<0.001

REM, random-effects model; FEM, fixed-effects model.

### Prognostic value of CRP for PFS

**Figure 3:**
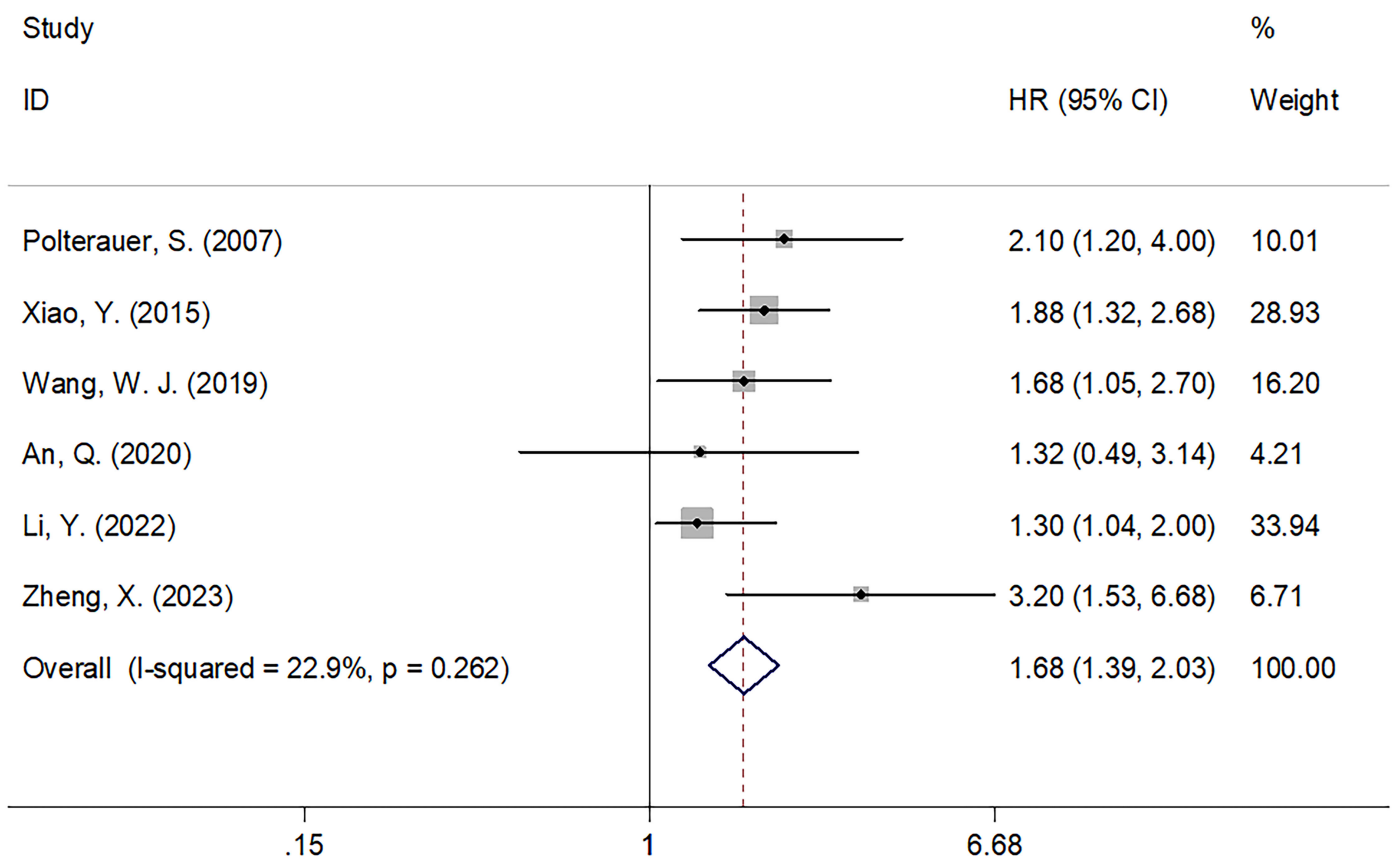
Forest plots of the prognostic role of CRP for PFS in patients with CC.

Of the 12 included articles, 6 studies, encompassing 1,415 patients (18, 20, 23, 24, 28, 29), provided data on the role of CRP values in predicting the PFS of patients with CC. The FEM was utilized with these articles, due to their low level of heterogeneity (I^2 = ^22.9%; *P* = 0.262; **Figure 3**). In line with our aforementioned analysis, however, increased CRP levels were significantly correlated with shortened PFS in patients with CC (HR = 1.68; 95% CI = 1.39–2.03; *P* < 0.001; **Figure 3**, **Table 3**). Similar to what we observed with the subgroup analysis for OS, an increased CRP level was significant in predicting PFS regardless of sample size, country, FIGO stage, treatment, CRP cut-off, cut-off determination, or survival analysis type (*P* < 0.05; **Table 3**).

**Table 3:** Subgroup analysis of prognostic role of CRP for PFS in patients with CC.

Subgroups	No. of studies	No. of patients	Effects model	HR (95%CI)	p	Heterogeneity I^2^(%) Ph	
						I^2^(%)	Ph
Total	6	1,415	FEM	1.68(1.39-2.03)	<0.001	22.9	0.262
Country							
China	5	1,201	FEM	1.64(1.34-2.01)	<0.001	32.3	0.206
Non-China	1	214	–	2.10(1.15-3.83)	0.016	–	–
Sample size							
<200	2	225	REM	2.18(1.17-4.04)	0.014	51.9	0.149
≥200	4	1,190	FEM	1.59(1.28-1.98)	<0.001	8.9	0.349
FIGO stage							
I-IV	2	452	FEM	1.93(1.43-2.62)	<0.001	0	0.756
I-II/I-III	3	848	FEM	1.41(1.09-1.82)	0.010	0	0.674
Recurrent/ metastatic	1	115	–	3.20(1.53-6.68)	0.002	–	–
Treatment							
CCRT	2	698	REM	1.55(1.08-2.23)	0.017	55.6	0.134
Mixed	3	602	FEM	1.75(1.24-2.47)	0.001	0	0.693
Anti-PD-1	1	115	–	3.20(1.53-6.68)	0.002	–	–
Cut-off value							
≤5 mg/L	4	899	FEM	1.64(1.29-2.05)	<0.001	48.0	0.123
>5 mg/L	2	516	FEM	1.80(1.29-2.50)	0.001	0	0.486
Cut-off determination							
ROC curve	4	1,091	FEM	1.63(1.31-2.04)	<0.001	49.1	0.117
Median value	2	324	FEM	1.83(1.26-2.66)	0.001	0	0.570
Survival analysis							
Multivariate	3	439	FEM	2.05(1.47-2.86)	<0.001	4.2	0.352
Univariate	3	976	FEM	1.53(1.21-1.93)	<0.001	14.9	0.309

REM, random-effects model; FEMl, fixed-effects model.

### Sensitivity analysis

**Figure 4:**
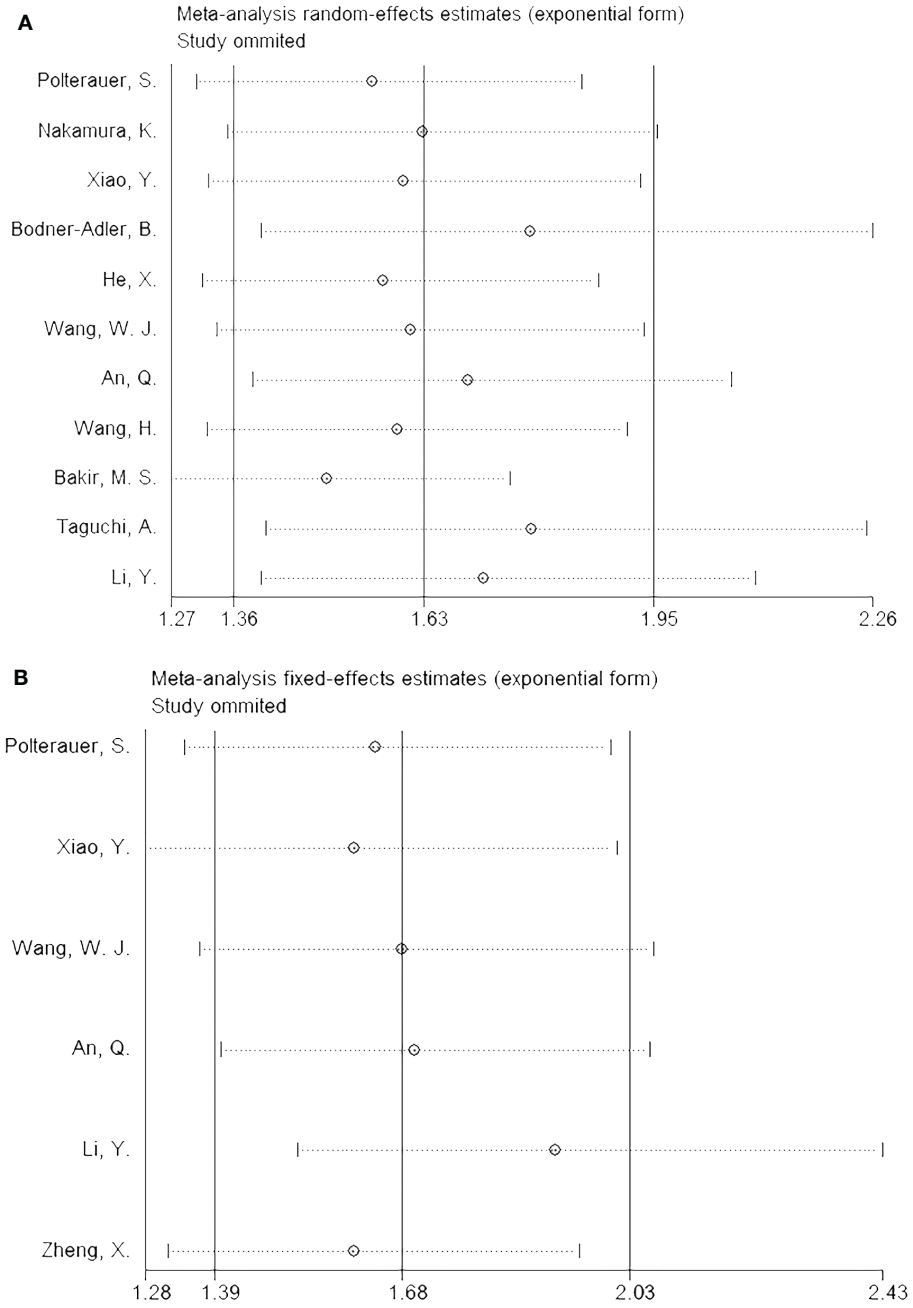
Sensitivity analysis. **(A)** OS; and **(B)** PFS.

The impact of each article on the combined results was analyzed by performing a sensitivity analysis, which revealed stable outcomes for both OS (**Figure 4A**) and PFS (**Figure 4B**).

### Publication bias

**Figure 5:**
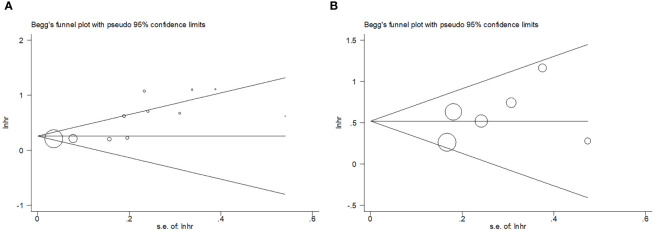
Publication test by Begg’ test. **(A)** Begg’s test for OS, p=0.082; **(B)** Begg’s test for PFS, p=0.452.

Begg’s test and funnel plots were used to assess publication bias. As shown in **Figure 5**, symmetry was observed in the funnel plots. For Begg’s test, the resulting P-value for OS was 0.082, while that for PFS was 0.452, indicating the absence of significant publication bias in the present study.

## Discussion

Although previous studies have evaluated the significance of CRP levels in determining the prognoses of patients with CC, the results were inconsistent. The present meta-analysis, which included 12 articles encompassing 2,204 cases, aimed to definitively identify the relationship of CRP levels with CC prognoses. Based on the findings of the present meta-analysis, increased serum CRP levels are significantly predictive of OS and PFS in patients with CC, and as demonstrated by the subgroup and sensitivity analyses, this relationship was very reliable. Taken together, these results suggest that serum CRP level may be a useful prognostic biomarker for patients with CC. To the best of our knowledge, this is the first meta-analysis to explore the prognostic significance of serum CRP levels in patients with CC.

CRP is a well-established biomarker for inflammation and can be easily assayed in clinical practice (42). Additionally, as an acute-phase protein generated by the liver, CRP is indicative of whether inflammation exists, as well as its level in the body, while maintaining certain advantages, such as a stable half-life, cost-effectiveness, simple measurement, and standardization (43). There are multiple mechanisms underlying the prognostic value of CRP in patients with CC. First, the presence of tumor tissue causes inflammation, which, in turn, increases serum CRP levels (44); therefore, increased CRP levels indicate tumor necrosis or local tissue injury. Second, IL-6 can induce hepatocytes to produce CRP, as can other factors, such as IL-1, TNF-α, and transforming growth factor β (TGFβ) (45), while via vascular endothelial growth factor (VEGF), IL-6 may promote angiogenesis in the presence of inflammation (46). Third, it has been shown that CRP induces an inflammatory tumor microenvironment that activates certain signaling pathways important for tumor proliferation, angiogenesis, and metastasis (47). It is likely, therefore, that patients with CC and a systemic inflammatory response, as indicated by elevated serum CRP levels, will be given a poor prognosis.

The present meta-analysis has several strengths. To the best of the authors’ knowledge, this is the first meta-analysis to explore the relationship between CRP levels and survival outcomes in patients with CC. Second, all included studies were of high quality (NOS score ≥ 6), which contributes to the overall statistical power of this meta-analysis. Third, sensitivity analyses and publication bias tests confirmed the reliability of the results of the present meta-analysis.

Numerous recent meta-analyses have reported the use of CRP in determining the prognoses of a variety of solid tumors. In a meta-analysis of 12 articles, Liu et al. (48) reported that serum CRP levels predicted poor OS in patients with prostate cancer. Li et al. (49) demonstrated that an increased CRP level indicated poor OS in patients with bone tumors, based on a meta-analysis of 816 participants. The results of a meta-analysis of 44 studies indicated that CRP levels could be used to predict OS and cancer-specific survival (CSS) in urological cancer cases (50). Jin et al. (51) carried out a meta-analysis encompassing 1,649 patients, the results of which showed that elevated CRP levels were indicative of poor OS in patients with non-small cell lung cancer, while Zheng et al. (52) reported that increased serum CRP levels indicated poor OS and recurrence-free survival (RFS) in patients with hepatocellular carcinoma, based on a meta-analysis of 10 studies. Notably, due to the fact that CRP is not specific for patients with CC. The level of CRP, as discussed above, could also offer insight into the prognosis of various types of cancer. In this case, the high level of CRP could serve as a prognostic marker only for those patients with CC without other cancers and treatment histories.

The present meta-analysis does have some limitations. First, the included studies were all of a retrospective nature, which may have introduced selection bias. Second, the cut-off values of CRP levels ranged from 2.6–10, but we were unable to determine an optimal cut-off value for determining the prognosis of CC based on CRP levels. Third, our sample size was relatively small, as many of the included studies had relatively small sample sizes. Therefore, larger prospective studies are required to verify our findings.

## Conclusions

In conclusion, the results of the present meta-analysis indicated that increased CRP levels are markedly associated with poor OS and PFS in patients with CC. Therefore, serum CRP level could be an inexpensive and independent factor for determining the prognoses of patients with CC in a clinical setting.

## Data availability statement

The original contributions presented in the study are included in the article/supplementary material. Further inquiries can be directed to the corresponding author.

## Author contributions

SY conceived and designed the study, interpreted the data, and drafted the manuscript. ZZ and LS designed and revised the manuscript. SY and ZZ selected the articles. ZZ and LS retrieved the data. All authors contributed to the article and approved the submitted version.
